# Association of loneliness and social isolation with excess risk of cardiovascular events in people with obesity: a prospective cohort study

**DOI:** 10.7189/jogh.15.04241

**Published:** 2025-10-03

**Authors:** Ying Zhou, Rui Chen, Minzhi Xu, Yanhong Gong, Chengbin Liu, Mengdie Wang, Xiaoxv Yin

**Affiliations:** 1 Department of Social Medicine and Health Management, School of Public Health, Tongji Medical College, Huazhong University of Science and Technology, Wuhan, Hubei, China; 2 School of Sociology, Huazhong University of Science and Technology, Wuhan, Hubei, China; 3 Department of Neurology, Union Hospital, Tongji Medical College, Huazhong University of Science and Technology, Wuhan, Hubei, China

## Abstract

**Background:**

Obese individuals face higher degrees of loneliness and social isolation, but evidence on the association between their levels of loneliness and social isolation and the excess risk of cardiovascular disease (CVD) is lacking. Our study aimed to explore the association between the level of loneliness and social isolation and the obesity-related excess risk of CVD events.

**Methods:**

We included 432 767 individuals from the UK Biobank at baseline (100 947 with obesity and 331 820 without obesity). The levels of loneliness and social isolation were defined by a two-item scale and a three-item scale at baseline, respectively. We ascertained CVD events, including coronary heart disease, stroke, heart failure, and CVD mortality, through linkage to inpatient records from primary and secondary health care settings and death registries.

**Results:**

A total of 29 767 non-obese and 14 312 obese participants developed incident CVD outcomes during a median (interquartile range (IQR)) follow-up of 12.48 (11.61, 13.22) years. The excess risk of CVD events, including CVD subtypes and CVD mortality, in obese people compared to non-obese people progressively decreased with lower baseline levels of loneliness and social isolation. Specifically, the excess risk of all CVD events decreased by 29% (for loneliness) and 15% (for social isolation). The excess risk of CVD mortality decreased by 25% (for loneliness) and 52% (for social isolation), respectively.

**Conclusions:**

Lower baseline levels of loneliness and social isolation were associated with lower obesity-related excess risk of CVD events. Our findings call for the integration of community engagement and social support networks into existing intervention programmes to provide more effective cardiovascular care for obese individuals.

There is widespread consensus in the scientific community that obesity ranks as one of the leading public health challenges of the 21st century [1]. Globally, 43% of adults were overweight and 16% were living with obesity [2]. It is reported that two-thirds of excess mortality in obese people is attributable to cardiovascular disease (CVD) [3], underscoring the critical and urgent need for CVD prevention in obese population.

The American Heart Association's framework on the social determinants of CVD includes ‘social support’ as a key domain [4]. Loneliness and social isolation are two core indicators of inadequate social support. Loneliness refers to a subjective emotional state that arises from perceived deficiencies in the quality of one's social relationships. Loneliness may activate the hypothalamic-pituitary-adrenal axis and sympathetic nervous system, leading to increased inflammation, immune system alterations, and vascular dysfunction, and elevating the risk of CVD [5]. Social isolation is an objective condition characterised by a lack of social contacts and interactions with family, friends, and wider community [6,7]. Epidemiologically, it is much more prevalent in urban settings of middle and high-income countries [8]. Social isolation does not necessarily lead to loneliness, but it may promote unhealthy behaviours due to the lack of social oversight. Moreover, socially isolated individuals may face barriers to accessing health care services, further exacerbating their vulnerability to CVD.

Obese people face much higher degrees of loneliness and social isolation than non-obese people, owing to worries about discrimination from social prejudice and the mobility inconvenience caused by large body size [9–11]. The association between loneliness/isolation and the risk of CVD has previously been demonstrated in the general population [12–15]. These explorations provide a basis for our study. To date, no studies have specifically focused on obese population. Our study sought to address this gap. We hypothesised that mitigating loneliness and social isolation can help reduce the excess obesity-related CVD risks. As loneliness and social isolation are modifiable psychological and social behavioural factors, validating this hypothesis may inform public health strategies aiming at alleviating the burdens of CVD.

In this prospective cohort study, we explored the association between the level of loneliness and social isolation and the excess risk of CVD events (including CVD subtypes and CVD mortality) among obese population, offering novel insights into the multifaceted determinants of CVD risk in this vulnerable population. Especially, we compared the relative contributions of loneliness and social isolation to those of other conventional risk factors in predicting adverse CVD events.

## METHODS

### Study population

The UK Biobank is a large-scale, population-based prospective cohort study that recruited over 500 000 adults aged 40–70 years between 2006 and 2010 across England, Scotland, and Wales. This cohort collected comprehensive data through a combination of touchscreen verbal interviews, questionnaires, bio-sample collection, and physical measurements.

From the initial cohort of 502 401 participants enrolled at baseline, we excluded individuals with missing data on loneliness, social isolation, body mass index (BMI), as well as those who had CVD history. After applying these exclusion criteria, 432 767 participants were included in the final analysis (Figure S1 in the **Online Supplementary Document**). Participants with a BMI of 30 or higher were classified as obese.

The UK Biobank was approved by the North West Multi-Centre Research Ethics Committee (No. 11/NW/0382), with all participants providing written informed consent.

### Definition of loneliness and social isolation

We referred to previous studies to measure loneliness and social isolation indices [7,16–18]. Loneliness was assessed by two baseline questionnaire items:

1) ‘Do you often feel lonely?’ and

2) ‘How often are you able to confide in someone close to you?’

These two items were modified from questions included in the validated revised UCLA Loneliness Scale (University of California, Los Angeles) [19,20]. Three baseline questionnaire items were used to evaluate levels of social isolation:

1) ‘Including yourself, how many people live in your household?’;

2) ‘How often do you visit friends or family or have them visit you?’;

3) ‘Which of the following leisure or social activities (sports club or gym, pub or social club, adult education class, religious group, or other group activities) do you engage in once a week or more often?’

These three items were drawn from similar questions in the validated Berkman-Syme social network index [21,22]. We categorised levels of loneliness and social isolation based on participants' responses to specific questionnaire items (Table S1 in the **Online Supplementary Document**) [16]. Loneliness was classified as index = 0 (no loneliness), index = 1 (mild), or index = 2 (severe). Social isolation was categorised as index = 0 (no isolation), index = 1 (mild), or index = 2–3 (severe).

### Ascertainment of CVD events

In the UK Biobank, disease information for all participants was obtained by linking inpatient records from health care settings and death registries. The primary outcome of this study was CVD events (incidence of and mortality from CVD). The second outcome was CVD subtypes, including coronary heart disease (CHD), stroke, heart failure, and CVD mortality. These diagnoses were coded according to the International Classification of Diseases, version 9th (ICD-9) and version 10th (ICD-10): CVD (ICD-9 = 410–414, 430–434, 436, 428; ICD-10 = I20-I25, I60-I64, I50), CHD (ICD-9 = 410–414; ICD-10 = I20-I25), stroke (ICD-9 = 430–434, 436; ICD-10 = I60-I64), heart failure (ICD-9 = 428; ICD-10 = I50), CVD mortality (ICD-10 = I00–I99) (Table S2 in the **Online Supplementary Document**) [23–28]. Follow-up duration was defined as the interval from the baseline date at recruitment until the first disease outcome diagnosis, death, loss to follow-up, or the termination of the follow-up (30 September 2021), whichever came first.

### Covariates

Confounding factors included sociodemographic factors, lifestyles, and health conditions (Table S3–5 in the **Online Supplementary Document**): age (continuous; years), sex (male or female), ethnicity (white British or others), Townsend deprivation index (continuous), education (college/university degree or others), diet (healthy or unhealthy), smoking status (non-current smoking or current smoking), alcohol consumption (moderate alcohol consumption or heavy alcohol consumption), exercise (sufficient or insufficient), C-reactive protein (continuous; mg/L), depression (yes or no), anxiety (yes or no), type 2 diabetes (yes or no), hypertension (yes or no), hyperlipidaemia (yes or no), and CVD family history (yes or no). A healthy diet was defined as appropriate intake of at least five of the ten dietary components (fruit, vegetables, fish, processed meats, unprocessed meats, whole grains, refined grains, vegetable oils, dairy, and sugar-sweetened beverages) [29,30]. Alcohol consumption of more than 14 g per day for women and more than 28 g per day for men is classified as heavy drinking [31]. Sufficient exercise was defined as ≥75 minutes of vigorous activity or ≥150 minutes of moderate activity per week.

### Statistical analyses

The baseline characteristics of participants, categorised by obesity status (obese and non-obese) and levels of loneliness and social isolation, were summarised using number (percentage) for categorical variables, mean (standard deviation (SD)) for normally distributed continuous variables, and median (interquartile range (IQR)) for non-normally distributed continuous variables. Group differences were assessed using χ^2^ test, ANOVA, and Kruskal-Wallis test, respectively.

In our study, we calculated both absolute and relative excess risk of CVD. We computed the absolute excess risk by calculating absolute rate differences per 1000 person-years of CVD, with 95% confidence intervals (CIs) estimated using Poisson regression [32]. This metric provides a direct measure of the absolute excess risk of CVD. Referencing established literature, we employed Cox proportional hazards models with non-obese participants as the reference group to calculate the association of varying degrees of loneliness and social isolation with excess relative risk of obesity-related CVD events (including incident CVD, CHD, stroke, heart failure, and CVD mortality) [16,33,34]. The follow-up duration was used as the time scale. Model 1 was unadjusted; model 2 was adjusted for age, sex, ethnicity, Townsend deprivation index, and education; model 3 was further adjusted for diet, smoking status, alcohol consumption, exercise, C-reactive protein, depression, anxiety, type 2 diabetes, hypertension, hyperlipidaemia, and CVD family history. Multiple imputation was employed to fill in missing covariates. We also drew the cumulative hazard of CVD events corresponding to loneliness/social isolation index and weight status.

In the subsequent analysis, we classified loneliness and social isolation into binary variables (0 or ≥1) and assessed the relative importance of loneliness and social isolation compared to other conventional risk factors (BMI, haemoglobin A1c (HbA_1c_), blood pressure, non-high-density lipoprotein (non-HDL), smoking status, sleep pattern, exercise, and diet) in predicting CVD events in obese population. The relative importance of these risk factors was measured by the R^2^ values of the Cox regression models, using coxphERR function of R software. The R^2^ essentially quantifies the proportion of independent contribution of a risk factor to the overall explanatory power of the model, reflecting the relative importance of that variable in explaining survival risk variation [35–37].

Interaction and subgroup analyses stratified by demographic characteristics were performed. We also conducted several sensitivity analyses. First, we treated death as a competitive event, and conducted competing risk analyses using Fine and Gray’s proportional sub-distribution hazards model. Second, we excluded participants with missing covariates to reduce the bias caused by imputation methods. Third, to minimise reverse causation, we excluded participants who encountered CVD events within two years from the baseline. Fourth, we used baseline blood pressure, blood glucose, and low-density lipoprotein cholesterol as proxies for hypertension, diabetes, and hyperlipidaemia.

All the statistical analyses were performed using *R*, version 4.3.1 (R Core Team, Vienna, Austria). Two-sided *P* less than 0.05 indicated statistically significant.

## RESULTS

### Baseline characteristics of participants

A total of 432 767 participants were included in this study, of whom 100 947 were obese (**Table 1**). Among people with obesity, 68 298 (67.7%), 26 513 (26.3%), and 6136 (6.1%) had a loneliness index of 0, 1, and 2, respectively; and 49 359 (48.9%), 40 975 (40.6%), and 10 613 (10.5%) had a social isolation index of 0, 1, and ≥2, respectively. The prevalence of both social isolation and loneliness was significantly higher in people with obesity than in those without obesity (*P* < 0.001) (Table S6 in the **Online Supplementary Document**).

**Table 1:** Baseline characteristics of participants

Variables*	No obesity (n = 331 820)	Obesity							
**Loneliness**				**Social isolation**			
**Index = 0 (n = 68 298)**	**Index = 1 (n = 26 513)**	**Index = 2 (n = 6136)**	***P*-value**	**Index = 0 (n = 49 359)**	**Index = 1 (n = 40 975)**	**Index ≥2 (n = 10 613)**	***P*-value**
Age, in years	49.0 (57.0, 63.0)	58.0 (51.0, 63.0)	57.0 (50.0, 62.0)	56.0 (49.0, 62.0)	<0.001	58.0 (50.0, 63.0)	57.0 (50.0, 63.0)	57.0 (51.0, 62.0)	<0.001
Female	187 635 (56.5%)	36 817 (53.9%)	15 058 (56.8%)	3247 (52.9%)	<0.001	25 820 (52.3%)	23 385 (57.1%)	5917 (55.8%)	<0.001
White British	296 790 (89.7%)	61 145 (89.8%)	23 076 (87.4%)	5353 (87.6%)	<0.001	44 061 (89.6%)	36 401 (89.1%)	9112 (86.3%)	<0.001
Townsend deprivation index	−3.8 (−2.3, 0.1)	−2.1 (−3.6, 0.6)	−1.3 (−3.2, 1.9)	−0.7 (−3.0, 2.4)	<0.001	−2.2 (−3.7, 0.3)	−1.5 (−3.3, 1.4)	−0.1 (−2.7, 3.0)	<0.001
College/university degree	120 245 (36.5%)	18 992 (28.0%)	5936 (22.6%)	1257 (20.7%)	<0.001	13 500 (27.6%)	10 109 (24.9%)	2576 (24.5%)	<0.001
Healthy diet	55 900 (18.1%)	9536 (15.0%)	3533 (14.4%)	789 (13.9%)	0.014	6696 (14.5%)	5628 (14.8%)	1534 (15.9%)	0.002
No current smoking	296 333 (89.5%)	62 295 (91.6%)	23 514 (89.0%)	5247 (85.9%)	<0.001	45 435 (92.3%)	36 635 (89.8%)	8986 (85.1%)	<0.001
Moderate alcohol consumption	241 790 (72.9%)	50 015 (73.3%)	19 999 (75.5%)	4636 (75.6%)	<0.001	34 308 (69.5%)	31 583 (77.1%)	8759 (82.6%)	<0.001
Sufficient physical activity	157 690 (57.0%)	26 249 (47.8%)	9192 (44.5%)	1886 (40.3%)	<0.001	20 801 (52.1%)	13 624 (42.4%)	2902 (35.3%)	<0.001
CRP, mg/L	0.6 (1.1, 2.1)	2.5 (1.4, 4.6)	2.7 (1.5, 5.1)	2.8 (1.5, 5.3)	<0.001	2.4 (1.3, 4.5)	2.6 (1.4, 5.0)	2.8 (1.5, 5.4)	<0.001
Type 2 diabetes	8177 (2.5%)	5880 (8.6%)	2743 (10.3%)	813 (13.2%)	<0.001	4037 (8.2%)	4038 (9.9%)	1361 (12.8%)	<0.001
Hypertension	70 298 (21.2%)	28 357 (41.5%)	11 435 (43.1%)	2824 (46.0%)	<0.001	20 092 (40.7%)	17 664 (43.1%)	4860 (45.8%)	<0.001
Hyperlipidaemia	39 997 (12.1%)	15 194 (22.2%)	6227 (23.5%)	1558 (25.4%)	<0.001	10 829 (21.9%)	9438 (23.0%)	2712 (25.6%)	<0.001
Depression	17 583 (5.3%)	3634 (5.3%)	3298 (12.4%)	1051 (17.1%)	<0.001	3186 (6.5%)	3475 (8.5%)	1322 (12.5%)	<0.001
Anxiety	5088 (1.5%)	919 (1.3%)	660 (2.5%)	171 (2.8%)	<0.001	751 (1.5%)	759 (1.9%)	240 (2.3%)	<0.001
CVD family history	182 226 (61.3%)	39 276 (64.7%)	15 307 (66.2%)	3558 (68.0%)	<0.001	28 540 (64.9%)	23 584 (65.4%)	6017 (66.8%)	0.002

CRP – C-reactive protein, CVD – cardiovascular disease

*Data are n (%) for categorical variables, mean (standard deviation) for normally distributed continuous variables, and median (interquartile range) for nonnormally distributed continuous variables.

### Loneliness and social isolation with excess risk of CVD events among obese people compared with non-obese people

We calculated the excess risk of CVD events in people with obesity at varying degrees of loneliness and social isolation, compared to people without obesity. During a median (IQR) follow-up of 12.48 (11.61, 13.22) years, 29 767 non-obese participants and 14 312 obese participants developed at least one incident CVD outcome.

**Figure 1:**
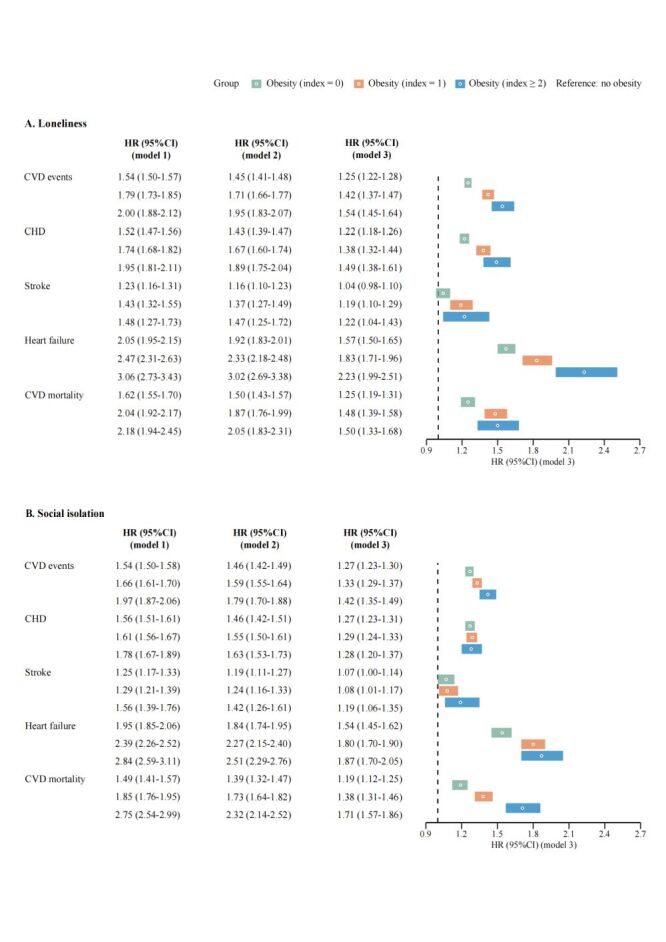
Association of loneliness (**Panel A**) and social isolation (**Panel B**) with excess risk of CVD events among obese people compared with non-obese people. Model 1 was unadjusted; model 2 was adjusted for age, sex, ethnicity, Townsend deprivation index, and education; model 3 was further adjusted for diet, smoking status, alcohol consumption, exercise, C-reactive protein, depression, anxiety, type 2 diabetes, hypertension, hyperlipidemia, and CVD family history. CI – confidence interval, CHD – coronary heart disease, CVD – cardiovascular disease, HR – hazard ratio.

**Figure 2:**
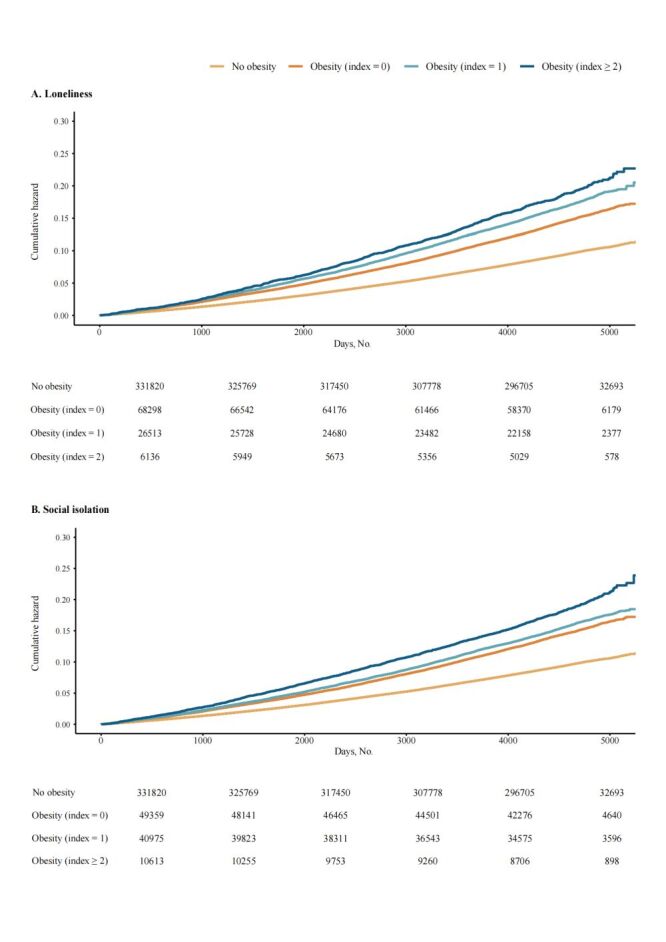
Cumulative hazard of cardiovascular events by weight status and loneliness (**Panel A**)/social isolation (**Panel B**) index.

Participants who were obese and with high levels of loneliness / social isolation have increased incidence rates of CVD events, with the absolute rate difference per 1000 person-years being 7.37 (95% CI = 6.47, 8.27) for loneliness and 7.12 (95% CI = 6.44, 7.80) for social isolation, respectively, compared to non-obese participants (**Table 2**). Obese individuals with lower baseline levels of loneliness (**Figure 1**, Panel A) and social isolation (**Figure 1**, Panel B) had attenuated excess CVD risk (including CVD subtypes and CVD mortality) compared to non-obese individuals. Specifically, obese individuals with the lowest (*vs*. highest) baseline loneliness levels showed 29% lower excess risk of CVD events and 25% lower CVD mortality risk (excess relative risk of CVD events for highest level = 54%; excess relative risk of CVD events for lowest level = 25%; excess relative risk of CVD mortality for highest level = 50%; excess relative risk of CVD mortality for lowest level = 25%), while those with the lowest (*vs*. highest) baseline social isolation levels showed 15% lower excess CVD event risk and 52% lower CVD mortality risk (excess relative risk of CVD events for highest level = 42%; excess relative risk of CVD events for lowest level = 27%; excess relative risk of CVD mortality for highest level = 71%; excess relative risk of CVD mortality for lowest level = 19%). Notably, when the degree of loneliness and social isolation was at its lowest, the risk of stroke among obese individuals was not statistically significantly different from that of non-obese individuals: loneliness (hazard ratio (HR) = 1.04; 95% CI = 0.98, 1.10, *P* = 0.196); social isolation (HR = 1.07; 95% CI = 1.00, 1.14, *P* = 0.052). However, given that the Cl is marginal, this result should be interpreted with caution. The cumulative hazard of CVD events concerning the loneliness (**Figure 2**, Panel A)/social isolation index (**Figure 2**, Panel B) and weight status is displayed. Additional details on the cumulative hazards for both incident CVD and CVD mortality (Figure S2–3 in the **Online Supplementary Document**), and associations of individual indicators of social contact and the joint effect of loneliness and social isolation with the risk of CVD events (Table S7–8 in the **Online Supplementary Document**).

**Table 2:** Incidence rate and absolute rate difference for CVD events among obese and non-obese people according to loneliness and social isolation level

Variables	Cases/n	Person-years	Incidence rate per 1000 (95% CI)	Absolute rate difference per 1000 (95% CI)
Loneliness				
*No obesity*	29 767 / 331 820	3 967 578	7.50 (7.42, 7.59)	-
*Obesity (index = 0)*	9164/68 298	797 850	11.49 (11.25, 11.72)	3.98 (3.73, 4.23)
*Obesity (index = 1)*	4100 / 26 513	306 644	13.37 (12.97, 13.78)	5.87 (5.45, 6.28)
*Obesity (index = 2)*	1048 / 6136	70 456	14.87 (14.00, 15.80)	7.37 (6.47, 8.27)
Social isolation				
*No obesity*	29 767 / 331 820	3 967 578	7.50 (7.42, 7.59)	-
*Obesity (index = 0)*	6661 / 49 359	578 350	11.52 (11.24, 11.80)	4.01 (3.73, 4.30)
*Obesity (index = 1)*	5882 / 40 975	475 648	12.37 (12.06, 12.69)	4.86 (4.54, 5.19)
*Obesity (index ≥ 2)*	1769 / 10 613	120 952	14.63 (13.96, 15.32)	7.12 (6.44, 7.80)

CI – confidence interval, CVD – cardiovascular disease

### Relative importance of loneliness and social isolation compared with traditional risk factors in predicting CVD events among obese participants

**Figure 3:**
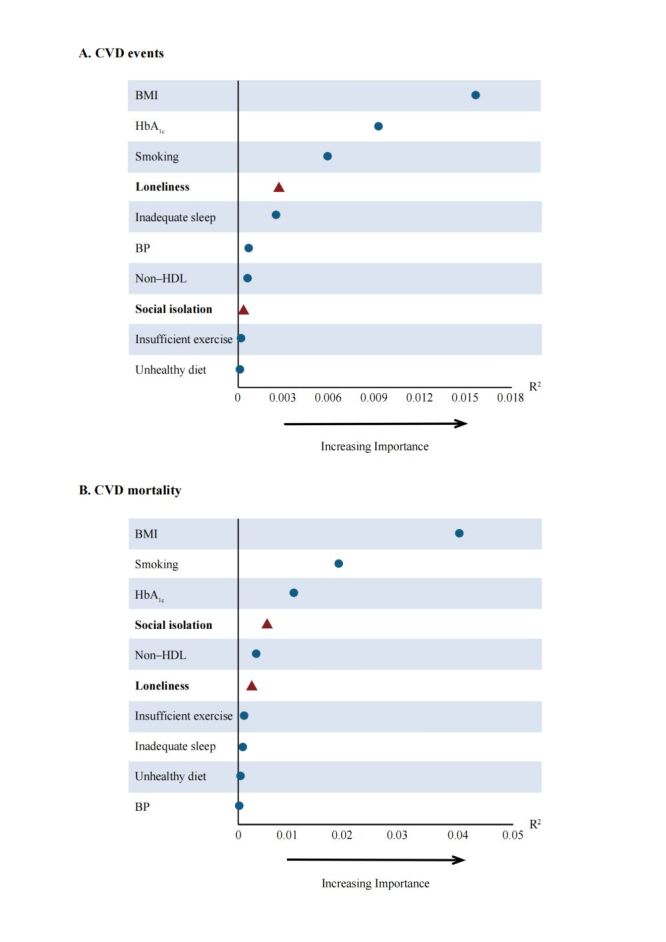
The relative importance of loneliness and social isolation compared with other traditional risk factors in predicting CVD events in obese people. BMI – body mass index, BP – blood pressure, CVD – cardiovascular disease, HbA_1c_ – haemoglobin A1c, Non-HDL – non-high-density lipoprotein.

We assessed the relative importance of loneliness and social isolation compared to other conventional risk factors (BMI, HbA_1c_, blood pressure, non-HDL, smoking status, sleep pattern, exercise, and diet) in predicting CVD events in participants with obesity. The results showed that loneliness and social isolation ranked fourth and eighth, respectively, in predicting all CVD events (**Figure 3**; Figure S4 in the **Online Supplementary Document**).

### Subgroup and sensitivity analysis

The associations between loneliness and CVD events in the obese population were significant across all demographic subgroups (Table S9 in the **Online Supplementary Document**). Significant interactions were found between loneliness and age (*P*_interaction_ = 0.027) and sex (*P*_interaction_ = 0.002). Specifically, the association between loneliness and risk of CVD events was stronger among obese people who were younger than 60 years and female. For social isolation, significant interactions were noted with sex (*P*_interaction_ = 0.001), Townsend deprivation index (*P*_interaction_ = 0.019), and education level (*P*_interaction_ = 0.041). The significant associations between social isolation and CVD events were limited to the female, relatively poorer, and less educated obese population. Four sensitivity analyses confirmed the robustness of our findings (Table S10–13 in the **Online Supplementary Document**).

## DISCUSSION

In this large population-based prospective cohort study, we observed that lower baseline levels of loneliness and social isolation were associated with attenuated excess risk of obesity-related CVD events. Obese individuals with the lowest (*vs*. highest) baseline loneliness levels showed 29% lower excess CVD risk, while those with the lowest (*vs*. highest) baseline social isolation levels showed 15% lower excess CVD risk. In addition, loneliness ranked fourth and social isolation ranked eighth among traditional risk factors in predicting CVD events.

Previous observational studies have discussed the relationship between social contact and CVD. A study that included 9235 Chinese adults observed that participants high increasing levels of loneliness had significantly increased risks of stroke and heart diseases compared to participants with stable low loneliness [38]. A cohort study from the USA found that loneliness and social isolation were associated with a higher risk of CVD among postmenopausal women [39]. A longitudinal study focused on older adults aged over 65 years, and found that those who reported feeling lonely had a 28% increased risk of developing CVD [40]. According to our knowledge, there are currently no studies specifically focusing on obese populations. Social isolation and loneliness are both more prevalent and more pronounced among obese individuals, who also bear a disproportionately higher burden of CVD morbidity. The absence of research in this area may lead to critical oversights in prevention and intervention strategies, thereby missing important opportunities to effectively mitigate CVD risk in this vulnerable population.

Our study found that obese individuals with the lowest (*vs*. highest) baseline levels of loneliness and social isolation had 29% and 15% lower excess risk of obesity-related CVD events, respectively. Considering the high prevalence of obesity worldwide, the observed associations suggest the potential importance of social isolation and loneliness in cardiovascular health, thereby carrying public health impact. Social isolation and loneliness are often associated with high levels of psychological stress, and stress can lead to elevated levels of cortisol in the body [5], which in turn affects blood pressure, blood glucose, and cholesterol levels [41–43], all of which are risk factors for CVD. Strong social relationships provide emotional support, helping individuals to better cope with life's challenges and reduce the incidence of negative emotions such as depression and anxiety [44], thereby reducing the burden placed on the cardiovascular system [45].

Furthermore, we compared the relative importance of social isolation and loneliness with other common CVD risk factors in predicting CVD. We found that, in predicting all CVD events, loneliness ranked fourth, following closely behind BMI, HbA_1c_, and smoking status. While traditional risk factors have been widely recognised, the impact of social determinants has often been underappreciated. Our study highlights the significant role that social connections play in the CVD risk among obese populations. These findings call for a more comprehensive approach to cardiovascular health, one that integrates social support networks into existing intervention programmes, to provide more effective cardiovascular care for obese individuals.

However, improving loneliness and social isolation remains a difficult task. Current interventions targeting social contact primarily include social activity-based interventions, psychological interventions, and technology-assisted interventions [46,47]. Few interventions have been specifically designed for obese populations. Obesity is often accompanied by diminished self-esteem and social stigmatisation, which may exacerbate social withdrawal and reduce willingness to participate. Therefore, interventions in this population require tailored modifications to traditional approaches. Key elements include fostering a safe, non-judgmental environment and helping obese individuals rebuild a sense of belonging in social settings. For instance, social activity-based interventions could incorporate body-inclusive programmes (*e.g.* film discussion, pet therapy) that shift focus from weight-related concerns to participatory experiences. In technology-assisted interventions, developing interest-based online platforms could facilitate virtual interactions, reducing anxiety related to physical exposure and thereby helping individuals rebuild social belonging in a secure and non-judgmental environment.

While prior research has established links between social contact and CVD, our study extends the existing evidence to obese populations – a group characterised by higher prevalence of social disconnection and greater cardiovascular vulnerability. The long follow-up time and substantial sample size in our research not only ensured a sufficient number of outcome events but also endowed our findings with robust statistical power and explanatory strength. Our study has several limitations. First, UK Biobank participants were more likely to be healthier and live in less socioeconomically deprived areas than nonparticipants [48], which could introduce selection bias. Second, nearly 90% of our study participants were White, which necessitates caution when generalising our findings to other ethnic groups. The association between social connections and CVD risk may vary across different ethnic backgrounds. Research showed that individuals from more interdependent cultures may exhibit stronger psychophysiological responses to interpersonal problems [49], potentially leading to a stronger link between social connectedness and CVD risk. Similarly, the association between obesity and CVD risk also shows heterogeneity across ethnic and socioeconomic groups. Body fat levels are higher in Asians compared with White Caucasians at similar BMI levels [50]. Importantly, obesity-related morbidities (such as CVD) occur more frequently at lower BMI levels in Asians than in White Caucasians [51]. Additionally, our study was conducted in the UK, whereas obese individuals living in underdeveloped areas often face multiple challenges, including limited access to health care resources and insufficient health education coverage [52], which may further increase their risk of CVD. These factors contributing to potential differences could not be fully explored due to the limitations of the UK Biobank study population’s diversity. Future multi-centre studies are needed to validate our findings in populations of diverse backgrounds. Third, our measures of loneliness and social isolation were based on brief self-reported items. Although these indicators were modified from validated scales, they may not fully capture the complexity of these constructs compared to multi-dimensional scales. Furthermore, the use of subjective questionnaires may be influenced by cultural differences [53], as perceptions of loneliness and social isolation can vary across different cultural contexts. Evidence suggested that people in collectivist cultures experience higher levels of loneliness than those in individualist cultures [54]. Collectivist societies place a high value on social connectedness, and a lack of social relationships can be experienced as painful. Therefore, when objective levels of isolation are comparable, people in collectivist cultures are more likely to feel lonely. This may limit the cross-cultural applicability of our findings. Fourth, loneliness and social isolation are dynamic factors that may evolve over time. However, our study assessed these variables only at baseline, limiting the ability to infer their real-time impact or changes on CVD outcomes. Future studies with repeated measurements of loneliness and social isolation are needed to better elucidate their temporal relationships with CVD risk. Finally, as an observational study, our research could not provide definitive evidence of a causal relationship between social contact and CVD events. While we observed associations between these factors, we could not establish a direct causality. The associations observed may be influenced by unmeasured or residual confounding factors (*e.g.* health care access disparities and cortisol levels). Additionally, reverse causation cannot be ruled out.

## CONCLUSIONS

This study found that lower baseline levels of loneliness and social isolation were associated with reduced obesity-related excess risk of CVD events. Loneliness and social isolation ranked fourth and eighth, respectively, among traditional risk factors in predicting CVD events. Our findings suggest the potential role of reducing loneliness and social isolation in lowering the excess CVD risk in individuals with obesity.

## Additional material


Online Supplementary Document
