# Supplementary material for: Association of loneliness and social isolation with excess risk of cardiovascular events in people with obesity: a prospective cohort study
Source: J Glob Health. 2025 Oct 3;15:04241. doi: 10.7189/jogh.15.04241 (PMC12491908; doi:10.7189/jogh.15.04241)
Supplement: Online Supplementary Document [file jogh-15-04241-s001.pdf]

Supplement to: Zhou Y, Chen R, Xu M, Gong Y, Liu C, Wang M, Yin X. Association of loneliness and social isolation with excess risk of cardiovascular events in people with obesity: a prospective cohort study. J Glob Health. 2025;15:04241.

Figure S1. Flow chart of the inclusion of participants 2
Figure S2. The cumulative hazard of CVD incidence and mortality corresponding to weight status and loneliness index 3
Figure S3. The cumulative hazard of CVD incidence and mortality corresponding to weight status and social isolation index 4
Figure S4. The relative importance of loneliness and social isolation compared with other traditional risk factors in predicting different subtypes of CVD in obese people 5
Table S1. Definition of loneliness and social isolation in the UK Biobank 6
Table S2. Definition of CVD in the UK Biobank 7
Table S3. Definitions of lifestyles in the UK Biobank 8
Table S4. Components of diet quality score used in the UK Biobank 9
Table S5. Definitions of diseases in the UK Biobank 11
Table S6. Distribution of loneliness and social isolation in the obese and non-obese participants 12
Table S7. Associations of individual indicators of loneliness and social isolation with the risk of CVD among obese people 13
Table S8. Joint effect of loneliness and social isolation with excess risk of CVD events among obese people 16
Table S9. Subgroup analyses of the associations between loneliness and social isolation and the risk of CVD in obese participants stratified by sociodemographic characteristics 17
Table S10. Associations of loneliness and social isolation with excess risk of CVD among obese people compared with non-obese people considering competing risk event 20
Table S11. Associations of loneliness and social isolation with excess risk of CVD among obese people compared with non-obese people after excluding patients who developed CVD within two years from baseline 22
Table S12. Associations of loneliness and social isolation with excess risk of CVD among obese people compared with non-obese people after excluding participants with missing covariates 24
Table S13. Associations of loneliness and social isolation with excess risk of CVD among obese people compared with non-obese people used baseline blood pressure, blood glucose and low-density lipoprotein cholesterol as proxies for hypertension, diabetes and hyperlipidemia 26

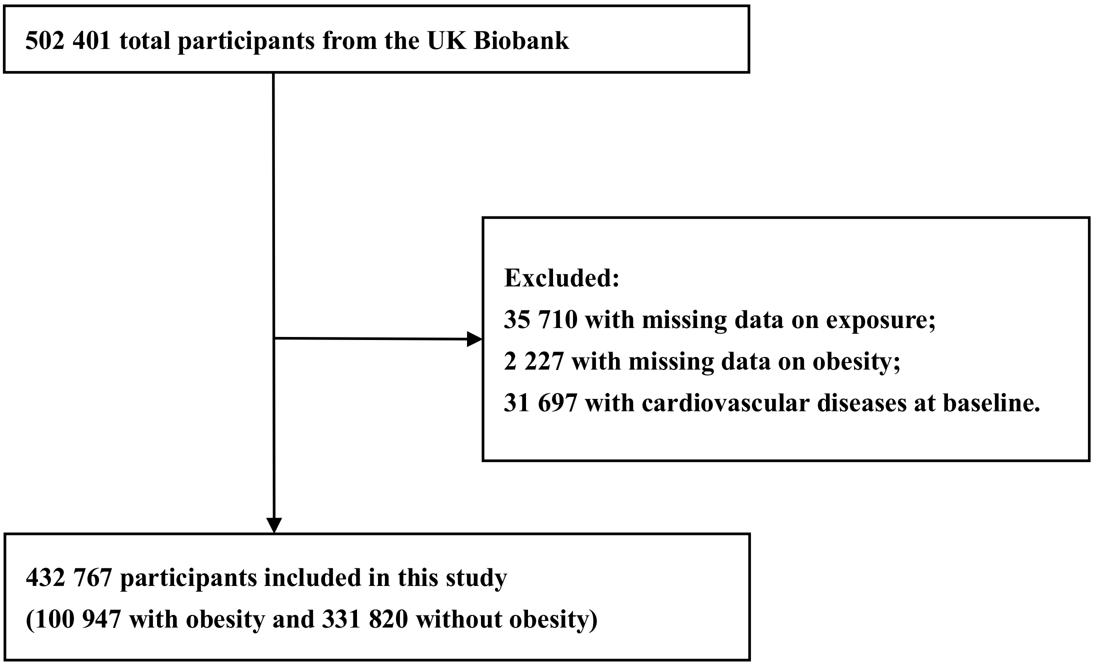

Figure S1. Flow chart of the inclusion of participants

A. CVD incidence

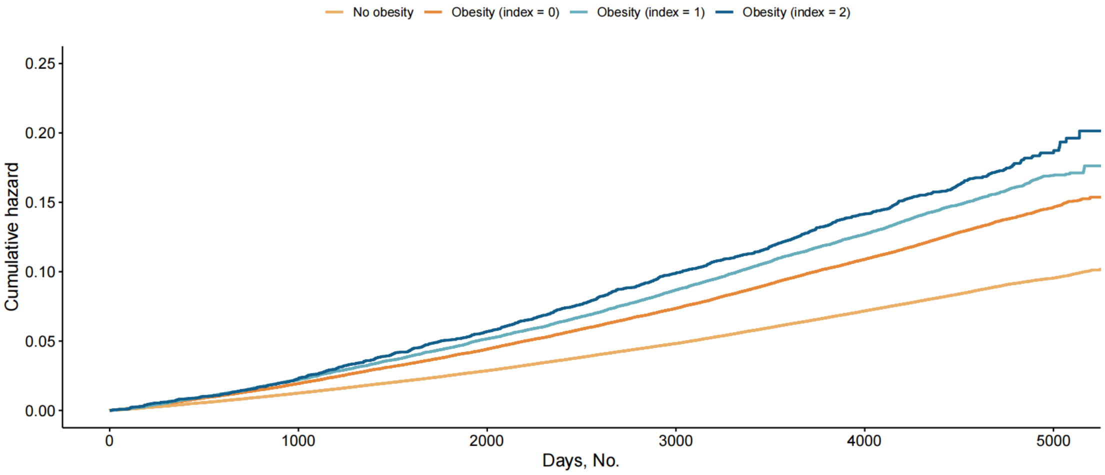

No obesity	331820	325769	317450	307778	296705	32693
Obesity (index = 0)	68298	66542	64176	61466	58370	6179
Obesity (index = 1)	26513	25728	24680	23482	22158	2377
Obesity (index = 2)	6136	5949	5673	5356	5029	578

B. CVD mortality

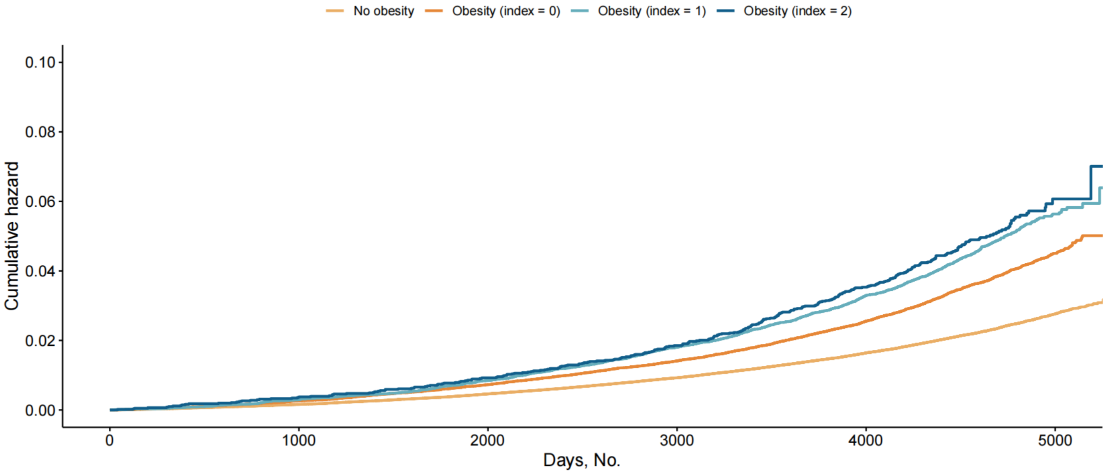

No obesity	331820	329672	326008	321545	316011	35159
Obesity (index = 0)	68298	67785	66875	65737	64265	6884
Obesity (index = 1)	26513	26273	25907	25410	24730	2702
Obesity (index = 2)	6136	6080	5985	5864	5700	652

Figure S2. The cumulative hazard of CVD incidence and mortality corresponding to weight status and loneliness index

Abbreviations: CVD, cardiovascular disease.

A. CVD incidence

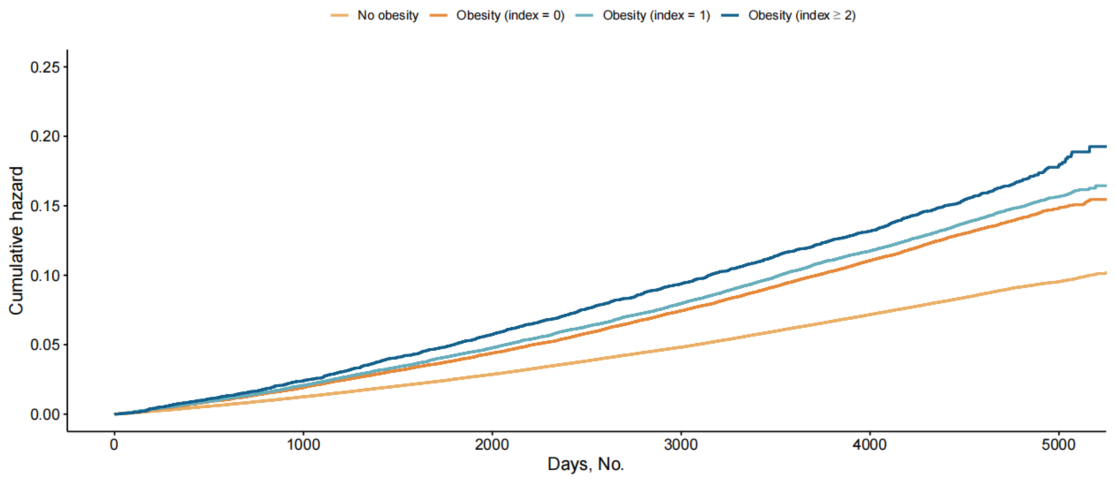

No obesity	331820	325769	317450	307778	296705	32693
Obesity (index = 0)	49359	48141	46465	44501	42276	4640
Obesity (index = 1)	40975	39823	38311	36543	34575	3596
Obesity (index ≥ 2)	10613	10255	9753	9260	8706	898

B. CVD mortality

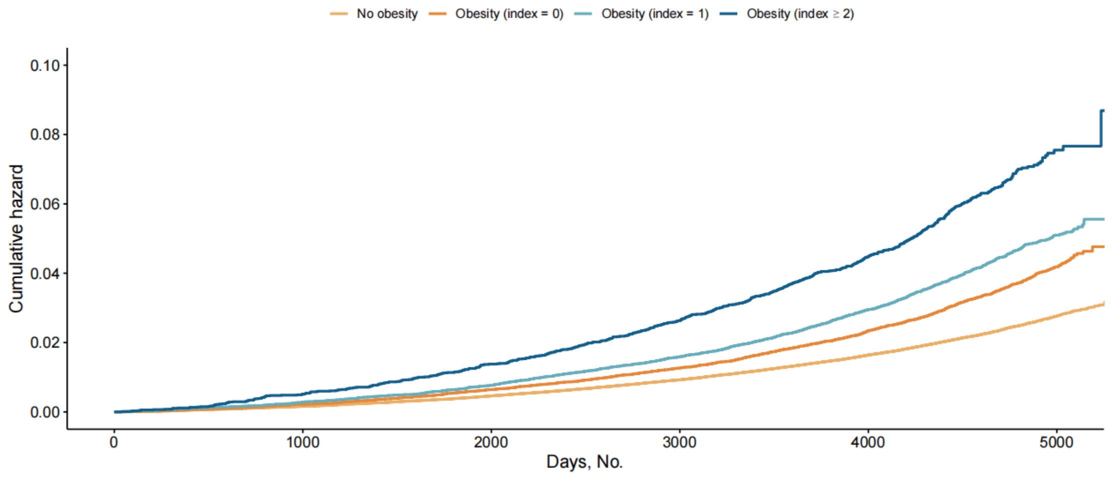

No obesity	331820	329672	326008	321545	316011	35159
Obesity (index = 0)	49359	49037	48425	47668	46674	5178
Obesity (index = 1)	40975	40616	40066	39293	38304	4033
Obesity (index ≥ 2)	10613	10485	10276	10050	9717	1027

Figure S3. The cumulative hazard of CVD incidence and mortality corresponding to weight status and social isolation index

Abbreviations: CVD, cardiovascular disease.

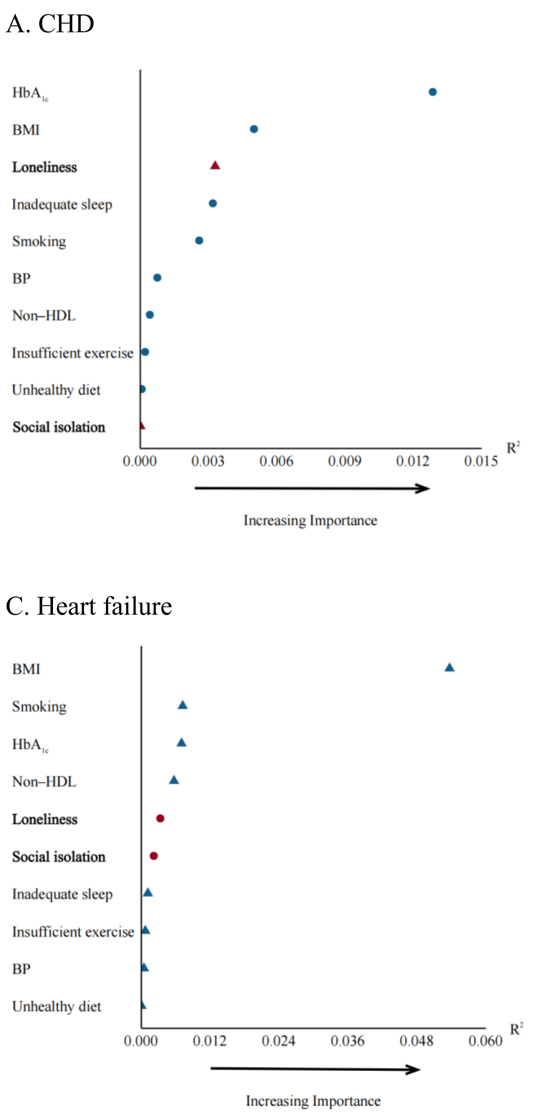

Figure S4. The relative importance of loneliness and social isolation compared with other traditional risk factors in predicting different subtypes of CVD in obese people

Abbreviations: BMI, body mass index; BP, blood pressure; CHD, coronary heart disease; CVD, cardiovascular disease; HbA_{1c}, hemoglobin A1c; Non-HDL, non-high-density lipoprotein.

Table S1. Definition of loneliness and social isolation in the UK Biobank

Data field IDs	ACE touchscreen questions	Responses	Score
Loneliness			
2020	Do you often feel lonely?	No	0
		Yes	1
2110	How often are you able to confide in someone close to you?	Almost daily	0
		2-4 times a week	0
		About once a week	0
		About once a month	0
		Once every few months	0
		Never or almost never	1
Social isolation			
709	Including yourself, how many people are living together in your household?	Not living alone	0
		Living alone	1
1031	How often do you visit friends or family or have them visit you?	Almost daily	0
		2-4 times a week	0
		About once a week	0
		About once a month	0
		Once every few months	1
		Never or almost never	1
		No friends/family outside household	1
6160	Which of the following (sports club or gym, pub or social club, religious group, adult education class, other group activity) do you attend once a week or more often?	Sports club or gym	0
		Pub or social club	0
		Religious group	0
		Adult education class	0
		Other group activity	0
		None of the above	1

Table S2. Definition of CVD in the UK Biobank

	ICD 9	ICD 10	Self-reported
Incident CVD	410-414, 430-434, 436, 428	I20-I25, I60-I64, I50	6150<br>20002
CHD	410-414	I20-I25	
Stroke	430-434, 436	I60-I64	
Heart failure	428	I50	
CVD mortality	-	I00-I99	

Abbreviations: CHD, coronary heart disease; CVD, cardiovascular disease.

Table S3. Definitions of lifestyles in the UK Biobank

Lifestyles	Definition
Diet	Healthy diet was defined as an adequate intake of at least 5 of the 10 dietary components (details were shown in Table S4)
Smoking status	The participants' current smoking status (current, previous, and never) was recorded, and no current smoking was on target.
Alcohol intake	Participants were asked about their drinking frequency and then further asked about the amount of red wine (glasses), champagne plus white wine (glasses), beer plus cider (pints), spirits (measures), fortified wine (glasses), and other alcoholic drinks (glasses) they consumed on average in a week or a month. Alcohol intake in units per day was calculated by summing the average individual drinks per day according to alcohol unit reference. The units were then converted to grams assuming that one unit equals 8 g. No more than 14 g/day for women and 28 g/day for men was on target.
Physical activity	The number of days of moderate/vigorous physical activity in a typical week and the duration of moderate/vigorous physical activity on a typical day were recorded. The number of days was multiplied by the time duration per day to calculate the weekly total amount of moderate or vigorous physical activity. Sufficient physical activity was defined as ≥150 minutes of moderate activity or ≥75 minutes of vigorous activity per week.

Detailed information on questionnaires can be obtained through the UK Biobank website (https://biobank.ndph.ox.ac.uk/showcase/).

Table S4. Components of diet quality score used in the UK Biobank

Components	Data field IDs	Amount per serving	Intake goal
Fruit	1309 (pieces fresh fruit/day)<br>1319 (pieces dried fruit/day)	1309: 1 piece<br>1319: 5 pieces	≥3 servings/day
Vegetable	1289 (tablespoons cooked vegetables/day)<br>1299 (salad/raw vegetables/day)	3 heaped tablespoons	≥3 servings/day
Fish	1329 (oily fish/week)<br>1339 (non-oily fish/week)	Once/week	≥2 servings/week
Processed meats	1349 (processed meat/week or daily)<br>3680 (age when last ate meat)	1349: 1 piece/day<br>3680: 0 pieces/day if indicated having never eaten meat	≤1 serving/week
Unprocessed red meats	1369 (beef/week or day)<br>1379 (lamb or mutton/week or day)<br>1389 (pork/week or day)<br>3680 (age when last ate meat)	1359~1389: once/week<br>3680: 0 pieces/day if indicated having never eaten meat	≤2 serving/week
Whole grains	1438, 1448 (whole meal/whole grain bread slices/week)<br>1458, 1468 (bran/oat/muesli cereal bowls/week)	1438/1448: 1 slice/day<br>1458/1468: 1bowl/day	≥3 servings/day
Refined grains	1438<br>1448 (white, brown, other bread slices/week)<br>1458<br>1468 (biscuit, other cereals/week)	1438/1448: 1 slice/day<br>1458/1468: 1bowl/day	≤2 servings/day
Vegetable oils	1428 (Flora Pro-Active/Benecol spread)<br>2654 (Flora Pro-Active/Benecol, soft margarine-, olive oil based-, polyunsaturated/sunflower oil based-, other low/reduced fat spread)<br>1438 (bread slices/week)	1 serving/day if in combination with eating at least 2 slices of bread (ID 1438)	≥2 servings/day
Dairy	1408 (cheese/week)<br>1418 (milk type)	1408: 1 piece/day<br>1418: 1 glass/day if consumption of anytype of	≥2 servings/day

Components	Data field IDs	Amount per serving	Intake goal
		milk	
Sugar-sweetened beverages	6144 (never consumes drinks containing sugar)	0 servings	Don't drink

Table S5. Definitions of diseases in the UK Biobank

Chronic diseases	Definition
Type 2 diabetes	Participants with type 2 diabetes were identified according to the algorithm developed by the UK Biobank. This algorithm takes into account multiple aspects of information such as self-reported medical history and medication, and has been proven valid with 96% accuracy.[1]
Hypertension	Participants with hypertension were identified according to ICD codes (ICD-9, 401-405; ICD-10, I10-I13, I15, O10), self-reported hypertension, or blood pressure medication.
Hyperlipidemia	Participants with hyperlipidemia were identified according to ICD codes (ICD-9, 272; ICD-10, E78), and self-reported information.
Depression	Participants with depression were identified according to ICD codes (ICD-9, 311; ICD-10, F32-F33).[2]
Anxiety	Participants with anxiety were identified according to ICD codes (ICD-9, 300; ICD-10, F40-F41).[2]

References

[1] Eastwood SV, Mathur R, Atkinson M, et al. (2016) Algorithms for the capture and adjudication of prevalent and incident diabetes in UK Biobank. PLOS ONE 11: e162388

[2] Gao X, Geng T, Jiang M, et al. Accelerated biological aging and risk of depression and anxiety: evidence from 424,299 UK Biobank participants. Nat Commun. 2023 Apr 20;14(1):2277.

Table S6. Distribution of loneliness and social isolation in the obese and non-obese participants

	Obesity (N = 100947)	No obesity (N = 331820)	*P* value
Loneliness			< 0.001
Index = 0	68298 (67.66%)	244669 (73.74%)	
Index = 1	26513 (26.26%)	73519 (22.16%)	
Index = 2	6136 (6.08%)	13632 (4.11%)	
Social isolation			< 0.001
Index = 0	49359 (48.90%)	180528 (54.41%)	
Index = 1	40975 (40.59%)	123562 (37.24%)	
Index ≥ 2	10613 (10.51%)	27730 (8.36%)	

Table S7. Associations of individual indicators of loneliness and social isolation with the risk of CVD among obese people

	Cases/Person-Years	HR (95%CI)
**CVD events**		
Items of loneliness		
Often feel lonely		
No	8139/680408	Ref.
Yes	2493/184664	1.22 (1.17-1.28)
Able to confide		
Once a month or more	8590/725127	Ref.
Less than once a month	2042/139944	1.06 (1.01-1.12)
Items of social isolation		
Live alone		
No	8234/698378	Ref.
Yes	2398/166694	1.06 (1.01-1.11)
Contact with family/friends		
Once a month or more	9664/791998	Ref.
Less than once a month	968/73074	1.05 (0.98-1.13)
Engaging in group activities once a week or more often		
Yes	6980/572604	Ref.
No	3652/292468	1.04 (1.00-1.09)
**CHD**		
Items of loneliness		
Often feel lonely		
No	5332/688395	Ref.
Yes	1672/187025	1.26 (1.19-1.33)
Able to confide		
Once a month or more	5680/733329	Ref.
Less than once a month	1324/142091	1.04 (0.98-1.10)
Items of social isolation		
Live alone		
No	5561/706183	Ref.
Yes	1443/169237	0.97 (0.91-1.03)
Contact with family/friends		
Once a month or more	6378/801409	Ref.
Less than once a month	626/74011	1.01 (0.93-1.10)
Engaging in group activities once a week or more often		
Yes	4637/579345	Ref.
No	2367/296075	1.01 (0.96-1.06)
**Stroke**		
Items of loneliness		
Often feel lonely		
No	1305/712155	Ref.
Yes	360/194620	1.10 (0.98-1.24)

	Cases/Person-Years	HR (95%CI)
Able to confide		
Once a month or more	1340/758677	Ref.
Less than once a month	325/148098	1.09 (0.97-1.23)
Items of social isolation		
Live alone		
No	1262/731460	Ref.
Yes	403/175315	1.15 (1.02-1.29)
Contact with family/friends		
Once a month or more	1525/829791	Ref.
Less than once a month	140/76984	1.00 (0.84-1.19)
Engaging in group activities once a week or more often		
Yes	1109/600104	Ref.
No	556/306671	1.03 (0.93-1.14)
**Heart failure**		
Items of loneliness		
Often feel lonely		
No	2129/710637	Ref.
Yes	699/193505	1.30 (1.19-1.42)
Able to confide		
Once a month or more	2242/756798	Ref.
Less than once a month	586/147345	1.14 (1.04-1.25)
Items of social isolation		
Live alone		
No	2109/729534	Ref.
Yes	719/174609	1.14 (1.04-1.25)
Contact with family/friends		
Once a month or more	2551/827657	Ref.
Less than once a month	277/76485	1.16 (1.02-1.31)
Engaging in group activities once a week or more often		
Yes	1833/598541	Ref.
No	995/305601	1.08 (1.00-1.17)
**CVD mortality**		
Items of loneliness		
Often feel lonely		
No	2174/717619	Ref.
Yes	665/195995	1.21 (1.10-1.32)
Able to confide		
Once a month or more	2244/764161	Ref.
Less than once a month	595/149452	1.11 (1.01-1.21)
Items of social isolation		
Live alone		
No	2033/736675	Ref.
Yes	806/176939	1.33 (1.22-1.45)

	Cases/Person-Years	HR (95%CI)
Contact with family/friends		
Once a month or more	2535/836086	Ref.
Less than once a month	304/77528	1.23 (1.09-1.38)
Engaging in group activities once a week or more often		
Yes	1800/604645	Ref.
No	1039/308968	1.15 (1.06-1.24)

Abbreviations: CHD, coronary heart disease; CI, confidence interval; CVD, cardiovascular disease; HR, hazard ratio.

Adjusted for age, sex, ethnicity, Townsend deprivation index, education, diet, smoking status, alcohol consumption, exercise, C-reactive protein, diabetes, hypertension, hyperlipidemia, depression, anxiety, and CVD family history.

Table S8. Joint effect of loneliness and social isolation with excess risk of CVD events among obese people

	Cases/Person-Years	HR (95%CI) (model 1)	HR (95%CI) (model 2)	HR (95%CI) (model 3)
**CVD events**				
No loneliness & No isolated	3687/322250	Ref.	Ref.	Ref.
No loneliness & Isolated	3177/269238	1.03 (0.99-1.08)	1.06 (1.01-1.11)	1.02 (0.97-1.07)
Loneliness & No isolated	1312/104635	1.10 (1.03-1.17)	1.16 (1.08-1.23)	1.12 (1.05-1.19)
Loneliness & Isolated	2456/168948	1.27 (1.21-1.34)	1.28 (1.22-1.35)	1.19 (1.13-1.26)
P value for trend		< 0.001	< 0.001	< 0.001
**CHD**				
No loneliness & No isolated	2486/325672	Ref.	Ref.	Ref.
No loneliness & Isolated	2038/272404	0.98 (0.92-1.04)	1.02 (0.96-1.08)	0.98 (0.92-1.04)
Loneliness & No isolated	905/105843	1.12 (1.04-1.21)	1.18 (1.09-1.27)	1.14 (1.06-1.23)
Loneliness & Isolated	1575/171501	1.21 (1.13-1.28)	1.23 (1.15-1.31)	1.14 (1.07-1.22)
P value for trend		< 0.001	< 0.001	< 0.001
**Stroke**				
No loneliness & No isolated	592/337057	Ref.	Ref.	Ref.
No loneliness & Isolated	496/281255	1.01 (0.89-1.13)	1.04 (0.92-1.17)	1.02 (0.90-1.15)
Loneliness & No isolated	196/109901	1.01 (0.86-1.19)	1.08 (0.92-1.27)	1.05 (0.89-1.23)
Loneliness & Isolated	381/178563	1.22 (1.07-1.39)	1.24 (1.08-1.41)	1.17 (1.03-1.34)
P value for trend		0.007	0.003	0.029
**Heart failure**				
No loneliness & No isolated	905/336438	Ref.	Ref.	Ref.
No loneliness & Isolated	869/280616	1.16 (1.05-1.27)	1.18 (1.07-1.29)	1.12 (1.02-1.23)
Loneliness & No isolated	325/109570	1.10 (0.97-1.25)	1.18 (1.03-1.33)	1.13 (0.99-1.28)
Loneliness & Isolated	729/177519	1.54 (1.39-1.69)	1.52 (1.37-1.68)	1.38 (1.25-1.53)
P value for trend		< 0.001	< 0.001	< 0.001
**CVD mortality**				
No loneliness & No isolated	873/339432	Ref.	Ref.	Ref.
No loneliness & Isolated	913/283397	1.26 (1.15-1.38)	1.29 (1.18-1.42)	1.22 (1.11-1.34)
Loneliness & No isolated	310/110688	1.08 (0.95-1.23)	1.16 (1.02-1.32)	1.11 (0.98-1.27)
Loneliness & Isolated	743/180098	1.61 (1.46-1.78)	1.60 (1.45-1.77)	1.43 (1.29-1.58)
P value for trend		< 0.001	< 0.001	< 0.001

Abbreviations: CHD, coronary heart disease; CI, confidence interval; CVD, cardiovascular disease; HR, hazard ratio.

Model 1 was unadjusted; model 2 was adjusted for age, sex, ethnicity, Townsend deprivation index, and education; model 3 was further adjusted for diet, smoking status, alcohol consumption, exercise, C-reactive protein, diabetes, hypertension, hyperlipidemia, depression, anxiety, and CVD family history.

Table S9. Subgroup analyses of the associations between loneliness and social isolation and the risk of CVD in obese participants stratified by sociodemographic characteristics

	Loneliness		Social isolation	
	HR (95%CI)	*P* for interaction	HR (95%CI)	*P* for interaction
**CVD events**				
Age, years		0.027		0.382
< 60	1.19 (1.13-1.26)		1.08 (1.02-1.14)	
≥ 60	1.13 (1.08-1.19)		1.08 (1.03-1.13)	
Sex		0.002		0.001
Female	1.22 (1.16-1.29)		1.14 (1.08-1.20)	
Male	1.11 (1.06-1.16)		1.03 (0.99-1.08)	
Ethnicity		0.116		0.207
White British	1.15 (1.11-1.19)		1.07 (1.03-1.11)	
Others	1.24 (1.11-1.37)		1.13 (1.01-1.25)	
Townsend deprivation index		0.061		0.019
Low	1.12 (1.06-1.18)		1.04 (0.99-1.09)	
High	1.18 (1.13-1.24)		1.12 (1.07-1.17)	
Education		0.456		0.041
College/university degree	1.13 (1.04-1.22)		1.01 (0.94-1.09)	
Others	1.17 (1.12-1.21)		1.10 (1.06-1.14)	
**CHD**				
Age, years		0.032		0.175
< 60	1.18 (1.11-1.26)		1.04 (0.98-1.11)	
≥ 60	1.11 (1.05-1.18)		1.02 (0.96-1.07)	
Sex		0.003		0.006
Female	1.22 (1.15-1.31)		1.08 (1.01-1.15)	
Male	1.10 (1.04-1.16)		0.99 (0.94-1.04)	
Ethnicity		0.032		0.153
White British	1.13 (1.08-1.18)		1.02 (0.97-1.06)	
Others	1.29 (1.13-1.46)		1.09 (0.96-1.24)	
Townsend deprivation index		0.135		0.400
Low	1.11 (1.04-1.19)		1.01 (0.95-1.08)	
High	1.17 (1.11-1.24)		1.04 (0.98-1.10)	
Education		0.445		0.011
College/university degree	1.11 (1.01-1.23)		0.93 (0.85-1.02)	
Others	1.16 (1.10-1.21)		1.06 (1.01-1.11)	
**Stroke**				
Age, years		0.208		0.217
< 60	1.05 (0.91-1.21)		0.97 (0.84-1.12)	
≥ 60	1.21 (1.09-1.35)		1.11 (1.00-1.23)	
Sex		0.607		0.232
Female	1.12 (0.99-1.28)		1.13 (0.99-1.28)	
Male	1.18 (1.05-1.32)		1.00 (0.89-1.12)	

	Loneliness		Social isolation	
	HR (95%CI)	*P* for interaction	HR (95%CI)	*P* for interaction
Ethnicity		0.590		0.185
White British	1.14 (1.04-1.25)		1.03 (0.95-1.13)	
Others	1.25 (0.96-1.63)		1.28 (0.98-1.68)	
Townsend deprivation index		0.163		0.305
Low	1.07 (0.94-1.22)		1.00 (0.88-1.13)	
High	1.22 (1.08-1.37)		1.11 (0.99-1.25)	
Education		0.267		0.113
College/university degree	1.03 (0.85-1.26)		0.91 (0.76-1.09)	
Others	1.19 (1.08-1.31)		1.10 (1.00-1.21)	
**Heart failure**				
Age, years		0.025		0.181
< 60	1.32 (1.17-1.48)		1.24 (1.09-1.40)	
≥ 60	1.16 (1.07-1.26)		1.17 (1.08-1.27)	
Sex		0.181		0.360
Female	1.26 (1.14-1.40)		1.21 (1.09-1.34)	
Male	1.17 (1.07-1.28)		1.18 (1.08-1.29)	
Ethnicity		0.788		0.335
White British	1.21 (1.13-1.30)		1.18 (1.10-1.27)	
Others	1.14 (0.92-1.40)		1.27 (1.02-1.58)	
Townsend deprivation index		0.469		0.149
Low	1.17 (1.06-1.30)		1.13 (1.03-1.25)	
High	1.23 (1.13-1.34)		1.25 (1.14-1.36)	
Education		0.222		0.688
College/university degree	1.09 (0.93-1.29)		1.16 (0.99-1.34)	
Others	1.23 (1.14-1.33)		1.20 (1.11-1.29)	
**CVD mortality**				
Age, years		0.556		0.030
< 60	1.20 (1.07-1.35)		1.37 (1.22-1.55)	
≥ 60	1.21 (1.12-1.31)		1.24 (1.15-1.34)	
Sex		0.583		0.888
Female	1.23 (1.11-1.37)		1.27 (1.14-1.41)	
Male	1.20 (1.10-1.31)		1.29 (1.18-1.40)	
Ethnicity		0.605		0.265
White British	1.21 (1.13-1.29)		1.27 (1.18-1.36)	
Others	1.25 (1.01-1.55)		1.43 (1.14-1.79)	
Townsend deprivation index		0.229		0.018
Low	1.17 (1.05-1.30)		1.19 (1.08-1.31)	
High	1.24 (1.14-1.35)		1.37 (1.25-1.49)	
Education		0.931		0.250
College/university degree	1.20 (1.04-1.40)		1.40 (1.21-1.62)	
Others	1.21 (1.13-1.30)		1.25 (1.16-1.34)	

Abbreviations: CHD, coronary heart disease; CI, confidence interval; CVD, cardiovascular disease; HR, hazard ratio.

Adjusted for age, sex, ethnicity, Townsend deprivation index, education, diet, smoking status, alcohol consumption, exercise, C-reactive protein, diabetes, hypertension, hyperlipidemia, depression, anxiety, and CVD family history.

Table S10. Associations of loneliness and social isolation with excess risk of CVD among obese people compared with non-obese people considering competing risk event

	Cases/Person-Years	HR (95%CI) (model 1)	HR (95%CI) (model 2)	HR (95%CI) (model 3)
**CVD events**				
Loneliness				
No obesity	27005/3967578	Ref.	Ref.	Ref.
Obesity (index = 0)	8247/797850	1.52 (1.48-1.56)	1.43 (1.40-1.47)	1.27 (1.24-1.31)
Obesity (index = 1)	3669/306644	1.75 (1.69-1.81)	1.68 (1.62-1.74)	1.45 (1.40-1.50)
Obesity (index = 2)	932/70456	1.94 (1.81-2.07)	1.90 (1.78-2.03)	1.57 (1.47-1.68)
Social isolation				
No obesity	27005/3967578	Ref.	Ref.	Ref.
Obesity (index = 0)	6059/578350	1.54 (1.50-1.58)	1.46 (1.42-1.50)	1.31 (1.27-1.35)
Obesity (index = 1)	5272/475648	1.62 (1.58-1.67)	1.56 (1.52-1.61)	1.35 (1.31-1.40)
Obesity (index ≥ 2)	1517/120952	1.83 (1.74-1.92)	1.67 (1.59-1.76)	1.39 (1.32-1.47)
**CHD**				
Loneliness				
No obesity	19545/3996578	Ref.	Ref.	Ref.
Obesity (index = 0)	5965/806709	1.51 (1.46-1.55)	1.42 (1.38-1.46)	1.25 (1.21-1.29)
Obesity (index = 1)	2643/310723	1.73 (1.66-1.80)	1.66 (1.59-1.73)	1.41 (1.35-1.47)
Obesity (index = 2)	680/71523	1.93 (1.79-2.08)	1.87 (1.73-2.02)	1.52 (1.40-1.64)
Social isolation				
No obesity	19545/3996578	Ref.	Ref.	Ref.
Obesity (index = 0)	4440/584695	1.55 (1.50-1.60)	1.46 (1.41-1.51)	1.30 (1.26-1.34)
Obesity (index = 1)	3788/481620	1.60 (1.54-1.66)	1.54 (1.49-1.59)	1.31 (1.27-1.36)
Obesity (index ≥ 2)	1060/122640	1.74 (1.64-1.85)	1.60 (1.50-1.70)	1.30 (1.22-1.39)
**Stroke**				
Loneliness				
No obesity	5878/4078087	Ref.	Ref.	Ref.
Obesity (index = 0)	1477/833087	1.22 (1.16-1.30)	1.16 (1.09-1.22)	1.06 (1.00-1.12)
Obesity (index = 1)	662/322356	1.41 (1.30-1.53)	1.36 (1.25-1.47)	1.21 (1.12-1.32)
Obesity (index = 2)	159/74601	1.46 (1.25-1.71)	1.45 (1.24-1.69)	1.25 (1.06-1.46)
Social isolation				
No obesity	5878/4078087	Ref.	Ref.	Ref.
Obesity (index = 0)	1087/604524	1.24 (1.17-1.33)	1.18 (1.11-1.26)	1.09 (1.02-1.17)
Obesity (index = 1)	926/498107	1.28 (1.19-1.37)	1.23 (1.14-1.32)	1.10 (1.03-1.19)
Obesity (index ≥ 2)	285/127413	1.53 (1.36-1.72)	1.39 (1.23-1.57)	1.21 (1.07-1.37)
**Heart failure**				
Loneliness				
No obesity	5682/4082527	Ref.	Ref.	Ref.
Obesity (index = 0)	2359/831276	2.04 (1.94-2.14)	1.90 (1.81-2.00)	1.63 (1.56-1.72)
Obesity (index = 1)	1096/321312	2.44 (2.29-2.60)	2.30 (2.15-2.45)	1.90 (1.78-2.03)
Obesity (index = 2)	313/73996	3.02 (2.70-3.39)	2.98 (2.65-3.34)	2.34 (2.08-2.63)
Social isolation				

	Cases/Person-Years	HR (95%CI) (model 1)	HR (95%CI) (model 2)	HR (95%CI) (model 3)
No obesity	5682/4082527	Ref.	Ref.	Ref.
Obesity (index = 0)	1635/603326	1.95 (1.84-2.06)	1.84 (1.74-1.95)	1.60 (1.51-1.69)
Obesity (index = 1)	1639/496540	2.37 (2.24-2.50)	2.24 (2.12-2.37)	1.87 (1.76-1.98)
Obesity (index ≥ 2)	494/126718	2.78 (2.53-3.04)	2.45 (2.23-2.69)	1.94 (1.76-2.13)

Abbreviations: CHD, coronary heart disease; CI, confidence interval; CVD, cardiovascular disease; HR, hazard ratio.

Model 1 was unadjusted; model 2 was adjusted for age, sex, ethnicity, Townsend deprivation index, and education; model 3 was further adjusted for diet, smoking status, alcohol consumption, exercise, C-reactive protein, diabetes, hypertension, hyperlipidemia, depression, anxiety, and CVD family history.

Table S11. Associations of loneliness and social isolation with excess risk of CVD among obese people compared with non-obese people after excluding patients who developed CVD within two years from baseline

	Cases/Person-Years	HR (95%CI) (model 1)	HR (95%CI) (model 2)	HR (95%CI) (model 3)
**CVD events**				
Loneliness				
No obesity	26795/3964550	Ref.	Ref.	Ref.
Obesity (index = 0)	8203/796876	1.53 (1.50-1.57)	1.44 (1.41-1.48)	1.25 (1.22-1.28)
Obesity (index = 1)	3694/306228	1.80 (1.74-1.86)	1.72 (1.67-1.78)	1.44 (1.39-1.49)
Obesity (index = 2)	948/70356	2.02 (1.89-2.15)	1.97 (1.85-2.10)	1.57 (1.47-1.68)
Social isolation				
No obesity	26795/3964550	Ref.	Ref.	Ref.
Obesity (index = 0)	5982/577656	1.54 (1.50-1.58)	1.46 (1.42-1.50)	1.28 (1.24-1.31)
Obesity (index = 1)	5284/475048	1.66 (1.61-1.71)	1.60 (1.55-1.64)	1.34 (1.30-1.38)
Obesity (index ≥ 2)	1579/120756	1.96 (1.86-2.06)	1.78 (1.69-1.88)	1.43 (1.36-1.51)
**CHD**				
Loneliness				
No obesity	17269/3988746	Ref.	Ref.	Ref.
Obesity (index = 0)	5243/804109	1.51 (1.47-1.56)	1.43 (1.38-1.47)	1.23 (1.19-1.27)
Obesity (index = 1)	2321/309692	1.74 (1.67-1.82)	1.67 (1.60-1.74)	1.38 (1.32-1.45)
Obesity (index = 2)	602/71334	1.96 (1.81-2.13)	1.90 (1.75-2.07)	1.51 (1.39-1.64)
Social isolation				
No obesity	17269/3988746	Ref.	Ref.	Ref.
Obesity (index = 0)	3906/582972	1.55 (1.50-1.61)	1.46 (1.41-1.51)	1.27 (1.23-1.32)
Obesity (index = 1)	3340/479989	1.62 (1.56-1.68)	1.56 (1.50-1.62)	1.30 (1.25-1.35)
Obesity (index ≥ 2)	920/122174	1.76 (1.64-1.88)	1.61 (1.51-1.72)	1.28 (1.20-1.37)
**Stroke**				
Loneliness				
No obesity	5347/4050854	Ref.	Ref.	Ref.
Obesity (index = 0)	1303/824278	1.20 (1.13-1.28)	1.13 (1.07-1.21)	1.02 (0.96-1.09)
Obesity (index = 1)	596/318598	1.42 (1.31-1.55)	1.37 (1.26-1.49)	1.20 (1.10-1.31)
Obesity (index = 2)	147/73695	1.52 (1.29-1.79)	1.51 (1.28-1.78)	1.28 (1.08-1.51)
Social isolation				
No obesity	5347/4050854	Ref.	Ref.	Ref.
Obesity (index = 0)	975/597980	1.24 (1.16-1.32)	1.18 (1.10-1.26)	1.06 (0.99-1.14)
Obesity (index = 1)	810/492879	1.25 (1.16-1.35)	1.20 (1.12-1.30)	1.06 (0.99-1.15)
Obesity (index ≥ 2)	261/125711	1.59 (1.40-1.80)	1.46 (1.29-1.65)	1.24 (1.10-1.41)
**Heart failure**				
Loneliness				
No obesity	5219/4053919	Ref.	Ref.	Ref.
Obesity (index = 0)	2163/822392	2.06 (1.96-2.16)	1.92 (1.83-2.02)	1.59 (1.51-1.68)
Obesity (index = 1)	1017/317563	2.51 (2.34-2.68)	2.37 (2.21-2.53)	1.88 (1.75-2.02)
Obesity (index = 2)	282/73232	3.01 (2.67-3.40)	2.97 (2.63-3.35)	2.23 (1.97-2.51)

	Cases/Person-Years	HR (95%CI) (model 1)	HR (95%CI) (model 2)	HR (95%CI) (model 3)
Social isolation				
No obesity	5219/4053919	Ref.	Ref.	Ref.
Obesity (index = 0)	1522/596720	1.99 (1.88-2.10)	1.88 (1.77-1.99)	1.57 (1.48-1.67)
Obesity (index = 1)	1499/491183	2.39 (2.26-2.53)	2.27 (2.14-2.41)	1.82 (1.71-1.93)
Obesity (index ≥ 2)	441/125286	2.78 (2.52-3.06)	2.46 (2.23-2.71)	1.85 (1.68-2.05)
**CVD mortality**				
Loneliness				
No obesity	6633/4069740	Ref.	Ref.	Ref.
Obesity (index = 0)	2179/828922	1.62 (1.54-1.70)	1.49 (1.42-1.57)	1.26 (1.19-1.32)
Obesity (index = 1)	1068/320683	2.05 (1.92-2.19)	1.89 (1.77-2.02)	1.51 (1.41-1.61)
Obesity (index = 2)	259/74254	2.14 (1.89-2.43)	2.02 (1.78-2.29)	1.49 (1.31-1.69)
Social isolation				
No obesity	6633/4069740	Ref.	Ref.	Ref.
Obesity (index = 0)	1466/601461	1.50 (1.41-1.58)	1.40 (1.32-1.48)	1.20 (1.13-1.27)
Obesity (index = 1)	1492/495803	1.86 (1.76-1.96)	1.73 (1.64-1.83)	1.40 (1.32-1.48)
Obesity (index ≥ 2)	548/126596	2.69 (2.47-2.94)	2.28 (2.09-2.50)	1.70 (1.56-1.86)

Abbreviations: CHD, coronary heart disease; CI, confidence interval; CVD, cardiovascular disease; HR, hazard ratio.

Model 1 was unadjusted; model 2 was adjusted for age, sex, ethnicity, Townsend deprivation index, and education; model 3 was further adjusted for diet, smoking status, alcohol consumption, exercise, C-reactive protein, diabetes, hypertension, hyperlipidemia, depression, anxiety, and CVD family history.

Table S12. Associations of loneliness and social isolation with excess risk of CVD among obese people compared with non-obese people after excluding participants with missing covariates

	Cases/Person-Years	HR (95%CI) (model 1)	HR (95%CI) (model 2)	HR (95%CI) (model 3)
**CVD events**				
Loneliness				
No obesity	18900/2609278	Ref.	Ref.	Ref.
Obesity (index = 0)	5522/501040	1.53 (1.48-1.57)	1.45 (1.40-1.49)	1.28 (1.24-1.32)
Obesity (index = 1)	2322/182284	1.77 (1.69-1.85)	1.70 (1.62-1.77)	1.46 (1.40-1.53)
Obesity (index = 2)	569/39996	1.98 (1.82-2.15)	1.94 (1.78-2.11)	1.59 (1.46-1.73)
Social isolation				
No obesity	18900/2609278	Ref.	Ref.	Ref.
Obesity (index = 0)	4082/367516	1.54 (1.49-1.59)	1.45 (1.40-1.50)	1.29 (1.25-1.34)
Obesity (index = 1)	3388/287564	1.63 (1.58-1.70)	1.58 (1.52-1.64)	1.37 (1.32-1.42)
Obesity (index ≥ 2)	943/68240	1.92 (1.80-2.05)	1.79 (1.67-1.91)	1.48 (1.38-1.58)
**CHD**				
Loneliness				
No obesity	12509/2627873	Ref.	Ref.	Ref.
Obesity (index = 0)	3657/506169	1.52 (1.47-1.58)	1.44 (1.39-1.49)	1.25 (1.21-1.30)
Obesity (index = 1)	1520/184536	1.74 (1.65-1.83)	1.66 (1.58-1.76)	1.41 (1.34-1.49)
Obesity (index = 2)	389/40569	2.02 (1.83-2.24)	1.97 (1.78-2.18)	1.59 (1.44-1.76)
Social isolation				
No obesity	12509/2627873	Ref.	Ref.	Ref.
Obesity (index = 0)	2759/371170	1.56 (1.50-1.63)	1.47 (1.41-1.53)	1.29 (1.24-1.35)
Obesity (index = 1)	2216/290974	1.60 (1.53-1.68)	1.55 (1.48-1.63)	1.32 (1.26-1.38)
Obesity (index ≥ 2)	591/69130	1.80 (1.66-1.96)	1.68 (1.55-1.83)	1.37 (1.26-1.49)
**Stroke**				
Loneliness				
No obesity	3686/2680007	Ref.	Ref.	Ref.
Obesity (index = 0)	893/522591	1.25 (1.16-1.34)	1.19 (1.10-1.28)	1.08 (1.00-1.17)
Obesity (index = 1)	356/191272	1.36 (1.22-1.51)	1.32 (1.18-1.47)	1.18 (1.06-1.32)
Obesity (index = 2)	91/42377	1.56 (1.27-1.93)	1.56 (1.27-1.93)	1.34 (1.08-1.65)
Social isolation				
No obesity	3686/2680007	Ref.	Ref.	Ref.
Obesity (index = 0)	662/383569	1.26 (1.16-1.36)	1.20 (1.10-1.30)	1.10 (1.01-1.19)
Obesity (index = 1)	526/300832	1.27 (1.16-1.40)	1.24 (1.13-1.36)	1.12 (1.02-1.23)
Obesity (index ≥ 2)	152/71839	1.55 (1.32-1.82)	1.45 (1.23-1.71)	1.26 (1.07-1.48)
**Heart failure**				
Loneliness				
No obesity	3501/2683307	Ref.	Ref.	Ref.
Obesity (index = 0)	1369/521724	2.02 (1.90-2.15)	1.91 (1.79-2.03)	1.63 (1.53-1.74)
Obesity (index = 1)	586/190733	2.37 (2.17-2.59)	2.26 (2.07-2.47)	1.88 (1.72-2.06)
Obesity (index = 2)	166/42091	3.04 (2.61-3.56)	3.02 (2.58-3.53)	2.35 (2.00-2.75)

	Cases/Person-Years	HR (95%CI) (model 1)	HR (95%CI) (model 2)	HR (95%CI) (model 3)
Social isolation				
No obesity	3501/2683307	Ref.	Ref.	Ref.
Obesity (index = 0)	948/382918	1.90 (1.77-2.04)	1.80 (1.68-1.94)	1.56 (1.45-1.68)
Obesity (index = 1)	919/300053	2.36 (2.20-2.54)	2.27 (2.11-2.45)	1.90 (1.76-2.04)
Obesity (index ≥ 2)	254/71576	2.75 (2.43-3.13)	2.50 (2.20-2.84)	1.97 (1.73-2.24)
**CVD mortality**				
Loneliness				
No obesity	4466/2695001	Ref.	Ref.	Ref.
Obesity (index = 0)	1385/526200	1.59 (1.50-1.69)	1.49 (1.40-1.58)	1.28 (1.20-1.36)
Obesity (index = 1)	654/192697	2.05 (1.89-2.23)	1.92 (1.77-2.09)	1.60 (1.47-1.74)
Obesity (index = 2)	143/42735	2.02 (1.71-2.39)	1.94 (1.64-2.29)	1.47 (1.24-1.74)
Social isolation				
No obesity	4466/2695001	Ref.	Ref.	Ref.
Obesity (index = 0)	963/386096	1.51 (1.40-1.61)	1.41 (1.32-1.52)	1.23 (1.15-1.33)
Obesity (index = 1)	910/303104	1.82 (1.70-1.95)	1.73 (1.61-1.86)	1.44 (1.34-1.55)
Obesity (index ≥ 2)	309/72432	2.60 (2.32-2.92)	2.28 (2.03-2.56)	1.76 (1.56-1.98)

Abbreviations: CHD, coronary heart disease; CI, confidence interval; CVD, cardiovascular disease; HR, hazard ratio.

Model 1 was unadjusted; model 2 was adjusted for age, sex, ethnicity, Townsend deprivation index, and education; model 3 was further adjusted for diet, smoking status, alcohol consumption, exercise, C-reactive protein, diabetes, hypertension, hyperlipidemia, depression, anxiety, and CVD family history.

Table S13. Associations of loneliness and social isolation with excess risk of CVD among obese people compared with non-obese people used baseline blood pressure, blood glucose and low-density lipoprotein cholesterol as proxies for hypertension, diabetes and hyperlipidemia

	Cases/Person-Years	HR (95%CI) (model 1)	HR (95%CI) (model 2)	HR (95%CI) (model 3)
**CVD events**				
Loneliness				
No obesity	29767/3967578	Ref.	Ref.	Ref.
Obesity (index = 0)	9164/797850	1.54 (1.50-1.57)	1.45 (1.41-1.48)	1.36 (1.33-1.39)
Obesity (index = 1)	4100/306644	1.79 (1.73-1.85)	1.71 (1.66-1.77)	1.57 (1.52-1.63)
Obesity (index = 2)	1048/70456	2.00 (1.88-2.12)	1.95 (1.83-2.07)	1.73 (1.62-1.84)
Social isolation				
No obesity	29767/3967578	Ref.	Ref.	Ref.
Obesity (index = 0)	6661/578350	1.54 (1.50-1.58)	1.46 (1.42-1.49)	1.38 (1.34-1.42)
Obesity (index = 1)	5882/475648	1.66 (1.61-1.70)	1.59 (1.55-1.64)	1.47 (1.43-1.51)
Obesity (index ≥ 2)	1769/120952	1.97 (1.87-2.06)	1.79 (1.70-1.88)	1.57 (1.50-1.65)
**CHD**				
Loneliness				
No obesity	19545/3996578	Ref.	Ref.	Ref.
Obesity (index = 0)	5965/806709	1.52 (1.47-1.56)	1.43 (1.39-1.47)	1.35 (1.31-1.39)
Obesity (index = 1)	2643/310723	1.74 (1.68-1.82)	1.67 (1.61-1.74)	1.55 (1.49-1.62)
Obesity (index = 2)	680/71523	1.95 (1.81-2.11)	1.89 (1.75-2.04)	1.69 (1.57-1.83)
Social isolation				
No obesity	19545/3996578	Ref.	Ref.	Ref.
Obesity (index = 0)	4440/584695	1.56 (1.51-1.61)	1.46 (1.42-1.51)	1.40 (1.35-1.45)
Obesity (index = 1)	3788/481620	1.61 (1.56-1.67)	1.55 (1.50-1.61)	1.44 (1.39-1.49)
Obesity (index ≥ 2)	1060/122640	1.78 (1.67-1.89)	1.63 (1.53-1.73)	1.44 (1.36-1.54)
**Stroke**				
Loneliness				
No obesity	5878/4078087	Ref.	Ref.	Ref.
Obesity (index = 0)	1477/833087	1.23 (1.16-1.31)	1.16 (1.10-1.23)	1.07 (1.01-1.14)
Obesity (index = 1)	662/322356	1.43 (1.32-1.55)	1.37 (1.27-1.49)	1.25 (1.15-1.36)
Obesity (index = 2)	159/74601	1.48 (1.27-1.73)	1.47 (1.25-1.72)	1.30 (1.11-1.52)
Social isolation				
No obesity	5878/4078087	Ref.	Ref.	Ref.
Obesity (index = 0)	1087/604524	1.25 (1.17-1.33)	1.19 (1.11-1.27)	1.10 (1.03-1.18)
Obesity (index = 1)	926/498107	1.29 (1.21-1.39)	1.24 (1.16-1.33)	1.13 (1.05-1.22)
Obesity (index ≥ 2)	285/127413	1.56 (1.39-1.76)	1.42 (1.26-1.61)	1.25 (1.11-1.42)
**Heart failure**				
Loneliness				
No obesity	5682/4082527	Ref.	Ref.	Ref.
Obesity (index = 0)	2359/831276	2.05 (1.95-2.15)	1.92 (1.82-2.01)	1.78 (1.69-1.87)
Obesity (index = 1)	1096/321312	2.47 (2.31-2.63)	2.33 (2.18-2.48)	2.10 (1.97-2.25)

	Cases/Person-Years	HR (95%CI) (model 1)	HR (95%CI) (model 2)	HR (95%CI) (model 3)
Obesity (index = 2)	313/73996	3.06 (2.73-3.43)	3.01 (2.69-3.38)	2.63 (2.34-2.95)
Social isolation				
No obesity	5682/4082527	Ref.	Ref.	Ref.
Obesity (index = 0)	1635/603326	1.95 (1.85-2.06)	1.84 (1.74-1.95)	1.73 (1.64-1.83)
Obesity (index = 1)	1639/496540	2.39 (2.26-2.52)	2.27 (2.15-2.40)	2.06 (1.95-2.18)
Obesity (index ≥ 2)	494/126718	2.84 (2.59-3.11)	2.51 (2.29-2.76)	2.17 (1.98-2.39)
**CVD mortality**				
Loneliness				
No obesity	7366/4101457	Ref.	Ref.	Ref.
Obesity (index = 0)	2436/839101	1.62 (1.55-1.70)	1.50 (1.43-1.57)	1.39 (1.33-1.46)
Obesity (index = 1)	1185/324974	2.04 (1.92-2.17)	1.87 (1.76-1.99)	1.68 (1.58-1.79)
Obesity (index = 2)	294/75225	2.18 (1.94-2.45)	2.05 (1.82-2.31)	1.73 (1.54-1.95)
Social isolation				
No obesity	7366/4101457	Ref.	Ref.	Ref.
Obesity (index = 0)	1626/608859	1.49 (1.41-1.57)	1.39 (1.32-1.47)	1.31 (1.24-1.39)
Obesity (index = 1)	1662/502013	1.85 (1.76-1.95)	1.73 (1.64-1.82)	1.56 (1.48-1.65)
Obesity (index ≥ 2)	627/128429	2.75 (2.54-2.99)	2.32 (2.14-2.52)	1.95 (1.79-2.12)

Abbreviations: CHD, coronary heart disease; CI, confidence interval; CVD, cardiovascular disease; HR, hazard ratio.

Model 1 was unadjusted; model 2 was adjusted for age, sex, ethnicity, Townsend deprivation index, and education; model 3 was further adjusted for diet, smoking status, alcohol consumption, exercise, blood pressure, blood glucose, low-density lipoprotein cholesterol, depression, anxiety, and CVD family history.
